# Kv7-specific activators hyperpolarize resting membrane potential and modulate human iPSC-derived sensory neuron excitability

**DOI:** 10.3389/fphar.2023.1138556

**Published:** 2023-02-27

**Authors:** Mark Estacion, Shujun Liu, Xiaoyang Cheng, Sulayman Dib-Hajj, Stephen G. Waxman

**Affiliations:** ^1^ Department of Neurology and Center for Neuroscience and Regeneration Research, Yale University School of Medicine, New Haven, CT, United States; ^2^ Rehabilitation Research Center, Veterans Affairs Connecticut Healthcare System, West Haven, CT, United States

**Keywords:** pain, Nav1.7 channel, Kv7 channel, multi-electrode arrary, Kv7 targeted compounds

## Abstract

Chronic pain is highly prevalent and remains a significant unmet global medical need. As part of a search for modulatory genes that confer pain resilience, we have studied two family cohorts where one individual reported much less pain than other family members that share the same pathogenic gain-of-function Nav1.7 mutation that confers hyperexcitability on pain-signaling dorsal root ganglion (DRG) neurons. In each of these kindreds, the pain-resilient individual carried a gain-of-function variant in Kv7.2 or Kv7.3, two potassium channels that stabilize membrane potential and reduce excitability. Our observation in this molecular genetic study that these gain-of-function Kv7.2 and 7.3 variants reduce DRG neuron excitability suggests that agents that activate or open Kv7 channels should attenuate sensory neuron firing. In the present study, we assess the effects on sensory neuron excitability of three Kv7 modulators—retigabine (Kv7.2 thru Kv7.5 activator), ICA-110381 (Kv7.2/Kv7.3 specific activator), and as a comparator ML277 (Kv7.1 specific activator)—in a “human-pain-in-a-dish” model (human iPSC-derived sensory neurons, iPSC-SN). Multi-electrode-array (MEA) recordings demonstrated inhibition of firing with retigabine and ICA-110381 (but not with ML277), with the concentration-response curve indicating that retigabine can achieve a 50% reduction of firing with sub-micromolar concentrations. Current-clamp recording demonstrated that retigabine hyperpolarized iPSC-SN resting potential and increased threshold. This study implicates Kv7.2/Kv7.3 channels as effective modulators of sensory neuron excitability, and suggest that compounds that specifically target Kv7.2/Kv7.3 currents in sensory neurons, including human sensory neurons, might provide an effective approach toward pain relief.

## 1 Introduction

Chronic pain is highly prevalent and remains a significant unmet global medical need because current treatments are not effective (Nahin, 2017). The wide use of opioids has been followed by an epidemic of misuse and addiction in the United States and other countries (Humphreys et al., 2022). In a long (12 months) randomized clinical trial in veterans with chronic pain, opioids were not better than non-steroidal anti-inflammatory drugs (Krebs et al., 2018). Existing analgesics including currently available sodium channel blockers are sometimes effective, but their therapeutic utility is limited due to tolerability issues and off-target side effects (Alsaloum et al., 2020). Thus, there is urgent need for treatments for chronic pain that are both safe and effective.

With this as a background we have been searching for modulatory genes that confer pain resilience. As part of this program, we previously used whole-exome sequencing and Induced Pluripotent Stem Cell (iPSC) modeling to study two well-characterized kindreds with inherited erythromelalgia (IEM) due to gain-of-function Nav1.7 mutations. IEM produces a striking picture of severe pain, usually present in all carriers of these mutations. These two kindreds were unusual in that they each included an outlier individual with markedly attenuated pain despite carrying the same disease-causing mutation in Nav1.7 as another member of the kindred. Our analysis revealed a gain-of- function variant (Kv7.2-T730A) in KCNQ2, which encodes the voltage-gated potassium channel Kv7.2, in one of these pain-resilient individuals (Mis et al., 2019), and a gain-of- function variant (Kv7.3-D755N) in KCNQ3, which encodes voltage-gated potassium channel Kv7.3, in the second pair-resilient subject (Yuan et al., 2021). In native human sensory neurons, Kv7.2 and Kv7.3 channel subunits co-assemble to form a heteromeric channel responsible for producing potassium M-current conductance that regulates subthreshold excitability of sensory neurons (Wang et al., 1998; Brown and Passmore, 2009). The heteromeric complex of Kv7.2 and Kv7.3 channel subunits is open at resting membrane potentials, and functions as a brake to prevent high-frequency firing of action potentials that are associated with pain.

On the basis of the action of Kv7.2 and 7.3 in reducing the excitability of pain-signaling neurons, agents that can activate or open Kv7 channels would be expected to attenuate their firing, similar to the effect bestowed by KCNQ gain-of-function variants. Kv7 activators, including isoform selective molecules have been developed. Whereas retigabine is a-Kv7.2 thru Kv7.5 modulator with a potent pharmacological action on heteromeric Kv7.2/Kv7.3 and Kv7.3/Kv7.5 complexes (Orhan et al., 2012; Ostacolo et al., 2020), ICA-069673 (Provence et al., 2015; Wang et al., 2018) and QO58 (Zhang et al., 2013; Teng et al., 2016) are highly selective for Kv7.2/Kv7.3. In this study, we assess the effects of three Kv7 modulators—retigabine (Kv7.2 thru Kv7.5 activator), ICA-110381 (Kv7.2/Kv7.3 specific activator), and as a comparator ML277 (Kv7.1 specific activator) on excitability of pain-signaling sensory neurons in a “human pain-in-a-dish” model (Alsaloum and Waxman, 2022)., i.e., in human sensory neurons differentiated from iPSCs derived from individual patients with chronic pain due to IEM.

## 2 Materials and methods

### 2.1 Differentiation of human iPSC cell lines from characterized patients into sensory neurons

Patient informed consent was obtained in order to derive induced pluripotent stem cells (iPSC) and their use in genomic and functional studies. iPSCs were generated from blood samples of the proband (P129) and an unrelated individual (P303) using CytoTune-iPS 2.0 Sendai Reprogramming Kit (Thermo Fisher Scientific) at Yale Stem Cell Center (Yuan et al., 2021). Cells were screened for pluripotent stem cell markers and tested for normal karyotype before differentiation. iPSCs were cultured for at least 16 generations before the start of differentiation into sensory neurons (i.e., iPSC-SNs). The study was approved by the Yale Human Investigation Committee.

iPSCs were maintained and passaged using mTeSR plus medium (StemCell Technologies, Canada). Differentiation was initiated using a modified Chambers protocol with LSB and 3i inhibitors (Chambers et al., 2012; Young et al., 2014). Ten differentiations were performed during the course of these experiments. Differentiated neurons were maintained in Neurobasal Medium supplemented with N2/B27 GlutaMAX (Thermo Fisher Scientific) and four nerve growth factors [recombinant human β-nerve growth factor, brain-derived neurotrophic factor, glial-derived neurotrophic factor, and neurotrophin-3 (25 ng/mL; PeproTech) for 8 weeks before functional assessments.

### 2.2 MEA recording

MEA recordings were obtained as previously described (Mis et al., 2019). Briefly, following a full course of differentiation of human iPSC cells into sensory neurons, the neurons were dissociated and then either replated into the wells of an MEA multi-well plate or cryogenically frozen and stored for future use. Spontaneous firing activity of these neurons was assessed using a multi-well MEA system (Maestro, Axion Biosystems). A 48-well recording plate was used, with each well containing 16 low-noise individual embedded microelectrodes with integrated ground electrodes, forming an 4 × 4 recording grid of electrodes across a 1 × 1-mm area. Each electrode had a diameter of 30 µm with 200 µm center-to-center spacing between individual electrodes. The wells of the MEA plate were coated with Matrigel to facilitate reattachment of the sensory neurons. A drop of neuronal cell suspension is placed directly over the array of electrodes and allowed to settle before filling the well with maintenance media. The number of wells plated depends upon the number of neurons harvested with a target concentration of 10^5^ neurons per well. To provide environmental control during recordings, an ECmini (Axion Biosystems) unit was used to maintain CO_2_ concentrations around MEA cultures with a flow of premixed gas (5% CO_2_, 20% O_2_, balance nitrogen), and temperature was maintained at either 22°C (ambient), 33°C (skin), 37°C (core body) or 42°C (warm). A spike detection criterion of >6 standard deviations above background signal was used to distinguish action potentials from noise.

The effect of compounds on the spontaneous activity of the iPSC-derived sensory neurons was determined in a before and after protocol. For the purpose of statistical replicates, an individual well of the multi-well MEA plate was analyzed for total spike counts among all the electrodes of the well. Once a selected temperature had reached equilibrium, a recording period of 10 min was acquired. To better evaluate the effects of Kv7 modulators in a statistically robust manner, we will utilize the approaches as described by (18) which shows that transforming to the Log10 of total spikes or mean firing rate creates an extended range of linear behavior for experimental data divided into quantiles. In addition, they demonstrate that creating treatment groups that are balanced for average means facilitates statistical comparisons. The various wells of the MEA plate were recorded in the “pre” condition and then the wells were divided into groups of three or more with equal means. Wells with a noticeably lower activity were excluded from being part of a group resulting, on occasion, with groups with unequal numbers. The plate was then removed from the MEA instrument and brought to a laminar flow hood to apply selected compounds to each group. For the 48-well plates, the maintenance volume of media is 600 µl. Compounds were prepared as 3X of target final concentrations in media. Compound application was performed by first removing 200 µl of media from each well and then replacing with 200 µl of the 3X compound to restore the 600 µl total volume and result in the neurons in the well exposed to 1X final concentrations of the compound being tested. The MEA plate was then returned to the MEA instrument and once the target temperature was re-equilibrated, an additional 10 min recording period was acquired.

### 2.3 Current-clamp analysis

Recordings were obtained using an EPC-10 amplifier and the PatchMaster program (HEKA Elecktronik). Data were sampled at 4 kHz and filtered at 2.9 kHz with a low-pass Bessel filter. Patch pipette resistance was 2–3 MΩ, and series resistance was not compensated. The recordings were performed using DMEM/F12 media supplemented with 10 mM HEPES as the bath solution. Pipettes were filled with an intracellular solution containing the following (in mM): 140 KCl, 5 HEPES, 3 Mg-ATP, 0.5 EGTA, with pH adjusted to 7.4 with NaOH. Liquid junction potential (LJP) was not corrected. All the recordings were performed at room temperature. Data were analyzed using Fitmaster (HEKA Elektronik) and Origin (MicroCal Software).

Only iPSC-SNs with a membrane potential stable for tens of seconds were chosen for analysis. Resting membrane potential was determined immediately after switching into current-clamp mode as the mean membrane voltage in the absence of current stimulation. Current threshold was defined as the minimum amount of current necessary to trigger an AP and was determined by injecting depolarizing 200 ms current steps in 5 pA increments until an AP was triggered. Retigabine (10 µM) or vehicle control (0.1% DMSO) was applied using a perfusion system (AutoMate Scientific) that directs a flow of the solution over the field of cells using a quartz needle positioned *via* a mechanical micromanipulator. Once perfusion was initiated, the resulting RMP was recorded if it reached stability and current threshold was remeasured. Recorded data were processed offline using Fitmaster, GraphPad Prizm, and Excel. Unless otherwise stated, data are expressed as mean ± SEM. Analyses were performed with GraphPad Prizm. Statistical tests used for each individual dataset and exact *p* values are stated in the Results section.

### 2.4 Drug studies

The following Kv7 targeted compounds were used in this study. Retigabine (Kv7.2 thru Kv7.5 activator), ICA-110381 (Kv7.2/Kv7.3 specific activator), and ML277 (Kv7.1 specific activator) (Adooq Bioscience) were dissolved in dimethyl sulfoxide (DMSO) to make a 30-mM stock solution and stored at −20°C. Working dilutions of 1000x solutions were mixed fresh daily (e.g., a 3 mM DMSO dilution stock will be mixed with media at 1:1000 dilution to be applied to the cells as a 3 µM final solution and 0.1% DMSO vehicle. The vendor Adooq Biosciences clarified that their compound ICA-110381 was referred to as compound 16 in Table 2 in the paper by Amato et al. (2011) as an analog of ICA-069673. The activity towards Kv7.2/3 is slightly better than ICA-069673 (0.38 vs. 0.69 µM) but the specificity against Kv7.1 is not as good (15 vs. > 100 µM).

## 3 Results

**FIGURE 1:**
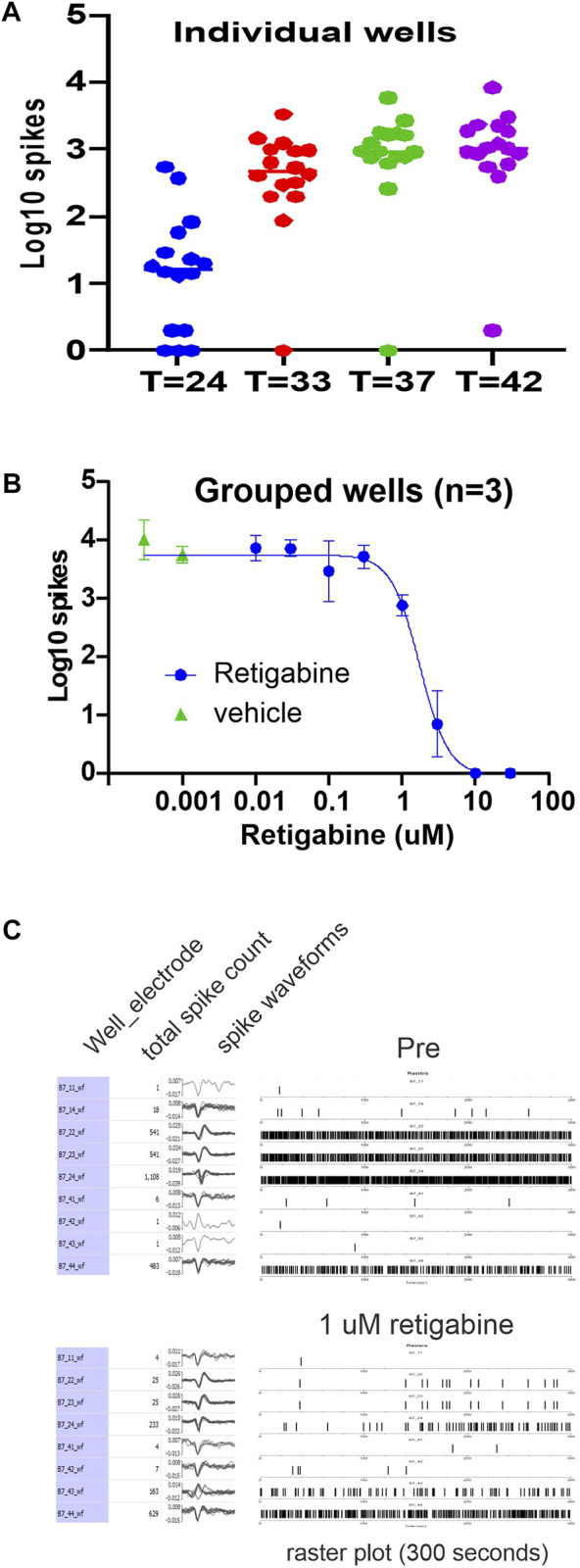
Retigabine reduces firing of IPSC-SNs in a dose-dependent manner. Spontaneous spiking activity of human iPSC-derived sensory neurons (clone 303) plated into the wells of a 48-well MEA plate. **(A)** The Log10 count of total spikes observed for a 10 min recording period is displayed with a symbol for each well. The panel illustrates the temperature response of the MEA activity and gives an indication of the well-to-well variability. The data represent the same plate recorded at the indicated temperatures in centigrade. **(B)** The panel shows the Log10 counts at 37°C that after group balancing, the dose-response relationship of the inhibition by retigabine is well determined with an IC_50_ of 1.7 µM. Retigabine is clearly effective at reducing spontaneous activity of the human iPSC-derived sensory neurons. **(C)** A display of the activity from a selected well that was exposed to 1 µM retigabine. In the pre-treatment recording, 9 of the 16 electrodes from well B7 detected spiking activity and the number of spikes, waveform of the spikes, and a raster plot of the timing of the spikes is shown. After exposure to 1 µM retigabine, 8 electrodes still recorded spiking activity.

We leveraged patient-specific iPSC-SNs to develop an *in vitro* platform that could be studied *via* non-invasive multi-electrode array assay to measure neuronal excitability of human sensory neurons in response to Kv7 modulators. The MEA system permits assessment of spontaneous action potentials and testing of novel pharmacological agents from intact human sensory neurons that have not been impaled by recording electrodes. Spontaneous activity of sensory neurons is a key driver of neuropathic pain, independent of an external stimulus (Devor, 2009). To establish a baseline, (Figure 1), we recorded spontaneous activity of iPSC-SN (clone 303) derived from a subject who did not have a pain syndrome, who did not carry any mutations in the ion channel genes known to be expressed in sensory neurons assessed by whole exome sequencing [clone 303, (Mis et al., 2019)] The sensory neurons, in addition to activity at ambient temperatures, clearly showed increasing activity levels as the temperature was elevated to physiological temperatures such as 33°C (skin temperature), 37°C (core body temperature) and 42°C (warm temperature) (Figure 1A). When the activity was transformed to the Log10 of total spikes observed during a 10-min recording period, the distribution of the values obtained from each well formed a distribution that is amenable to statistical characterization (Negri et al., 2020). Subdividing of the wells into smaller groups of three or more wells that maintain similar means (Negri et al., 2020) facilitates testing of pharmacological compounds that could modulate neuronal firing.

To assess modulation of spontaneous activity by Kv7 modulators in these iPSC-SNs, we first assessed retigabine, which is a Kv7.2 thru Kv7.5 activator and would be expected to modulate Kv7 channel subunits in any tissue where they are expressed. RNAseq of DRG neurons suggest that the predominant isoforms of Kv7 channels expressed are Kv7.2 and Kv7.3 (Hu et al., 2016; Hockley et al., 2019). For a representative experimental plate, which had 32-wells plated, ten groups were created which allowed the testing of eight concentrations of retigabine along with two groups of DMSO vehicle control. Analysis of the Log10 of total spikes obtained during a 10-min recording period at 37°C showed that retigabine was effective at reducing spontaneous spiking activity in a concentration-dependent manner. The resulting data were well fit with a logistic function resulting in an IC_50_ value of 1.7 µM. A display of the activity of a well before and after exposure to 1 µM retigabine is also shown (Figure 1C).

**FIGURE 2:**
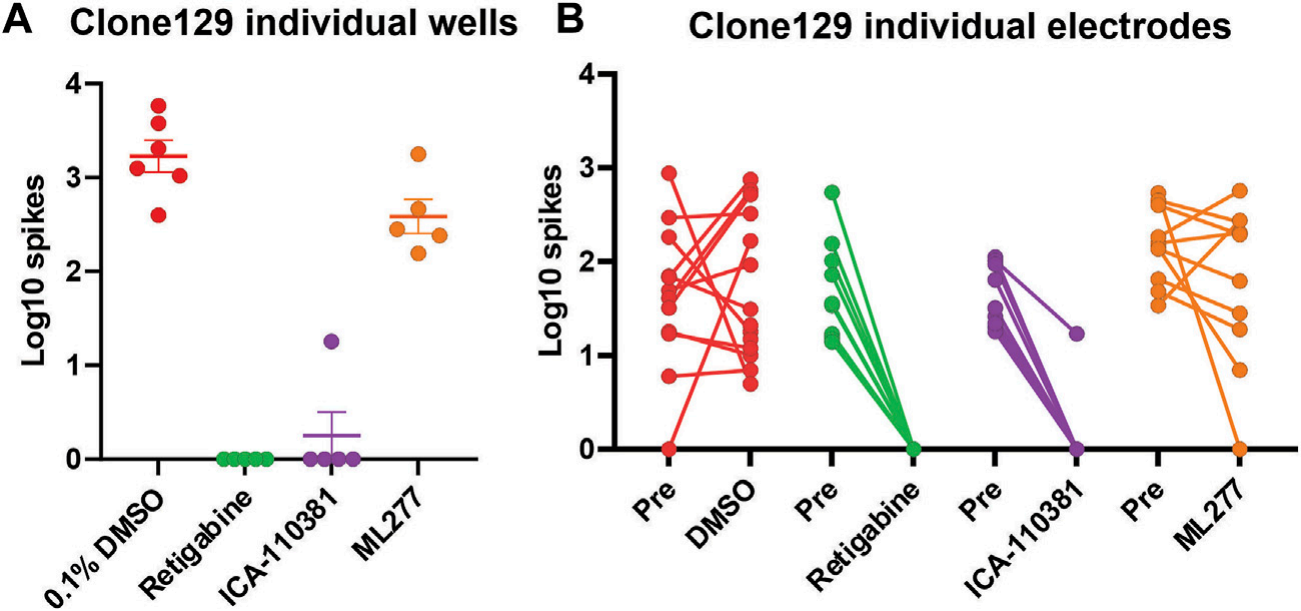
Relative response of Kv7 selective compounds. Spontaneous spiking activity of human iPSC-derived sensory neurons (clone 129) plated into the wells of a 48-well MEA plate. **(A)** The Log10 count of total spikes per well observed for a 10 min recording period at 37°C is displayed with a symbol for each well. The data groupings showed relatively equal means and SEM before treatment with the indicated compounds all at the same concentration of 3 µM. Both retigabine and ICA-110381 significantly reduced spontaneous spiking activity (*p* < 0.001) while ML277 showed a small but not significant reduction of activity (*p* = 0.54) compared to vehicle control. **(B)** The overall well responses were mirrored at the level of individual electrodes which likely represent the activity of individual sensory neurons as shown in the before and after plots.

We next studied the effect of Kv7 activators on hyperexcitable IPSC-derived sensory neurons from a subject with IEM. In these experiments we assessed iPSC-SN (clone 129) from a patient with IEM and severe pain due to a gain-of-function mutation (F1449V) in Nav1.7 (Dib-Hajj et al., 2005). This subject carries WT Kv7.2 and Kv7.3 subunits (Yuan et al., 2021). We assessed retigabine, a Kv7.2 thru Kv7.5 activator (Tatulian et al., 2001), ICA-110381, which is reported to be selective for Kv7.2/Kv7.3 subunits (Amato et al., 2011), and ML277, which in contrast, is reported to be specific for Kv7.1 subunits (Mattmann et al., 2012; Willegems et al., 2022). We compared the responses to these three compounds to iPSC-SN from clone 129. In this multi-well MEA plate, we were able to plate 24 wells and assessed the distribution of the Log10 spiking of the IPSC-SN and the assignment of the wells into four groups is shown (Figure 2A). Exposures to DMSO vehicle control as well as retigabine, ICA-110381 and ML277 at 3 µM final concentrations were assessed and clearly show that both the Kv7.2 thru Kv7.5 activator retigabine as well as the Kv7.2/Kv7.3 activator ICA-110381 profoundly inhibited spontaneous activity while the Kv7.1 activator ML277 showed little or no inhibition of spontaneous spiking (Figure 2B).

**FIGURE 3:**
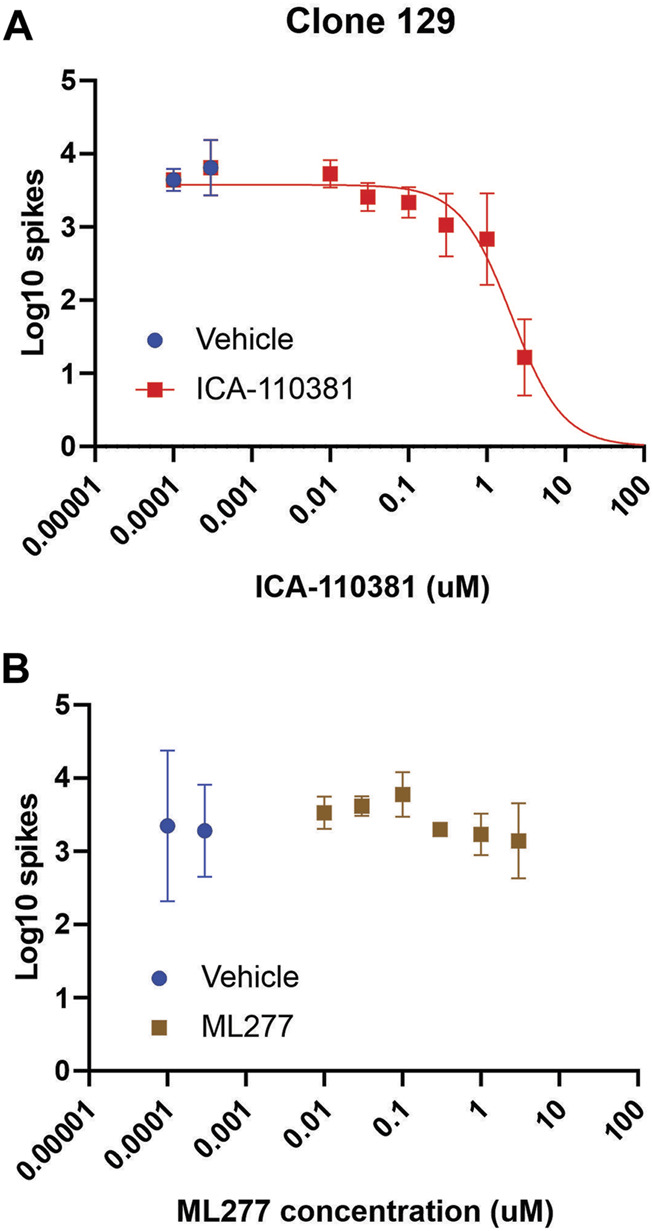
ICA-110381 a Kv7.2/Kv7.3 specific activator, attenuates firing of iPSC-SNs, while ML277, a Kv7.1 specific activator, does not Spontaneous spiking activity of human iPSC-derived sensory neurons (clone 129) plated into the wells of a 48-well MEA plate. **(A)** The panel shows the Log10 counts at 37°C that after group balancing, the dose-response relationship of the inhibition by ICA-110381 is well determined with an IC_50_ of 2.0 µM. ICA-110381 is clearly effective at reducing spontaneous activity of the human iPSC-derived sensory neurons. Spontaneous spiking activity of human iPSC-derived sensory neurons (clone 129) plated into the wells of a 48-well MEA plate. **(B)** The panel shows the Log10 counts at 37°C for balanced groups indicate that the inhibitory effect of ML277 at the chosen concentrations is minimal. The attempt to fit an inhibitory dose-response curve failed and thus is not shown.

We expanded on the characterization of the Kv7.2/Kv7.3 specific activator ICA-110381 and the Kv7.1 specific activator ML277 on the inhibition of spontaneous activity from the Nav1.7-F1449V expressing human iPSC-derived sensory neurons (clone 129) by performing eight-point dose-response recordings. The Kv7.2/Kv7.3 specific activator ICA-110381 exhibited a clear dose-response inhibition resulting in a logistic fit IC_50_ value of 2.0 µM (Figure 3A). The Kv7.1 specific activator, in contrast, did not significantly inhibit spontaneous spiking activity and no binding logistic fit was found (Figure 3B).

**FIGURE 4:**
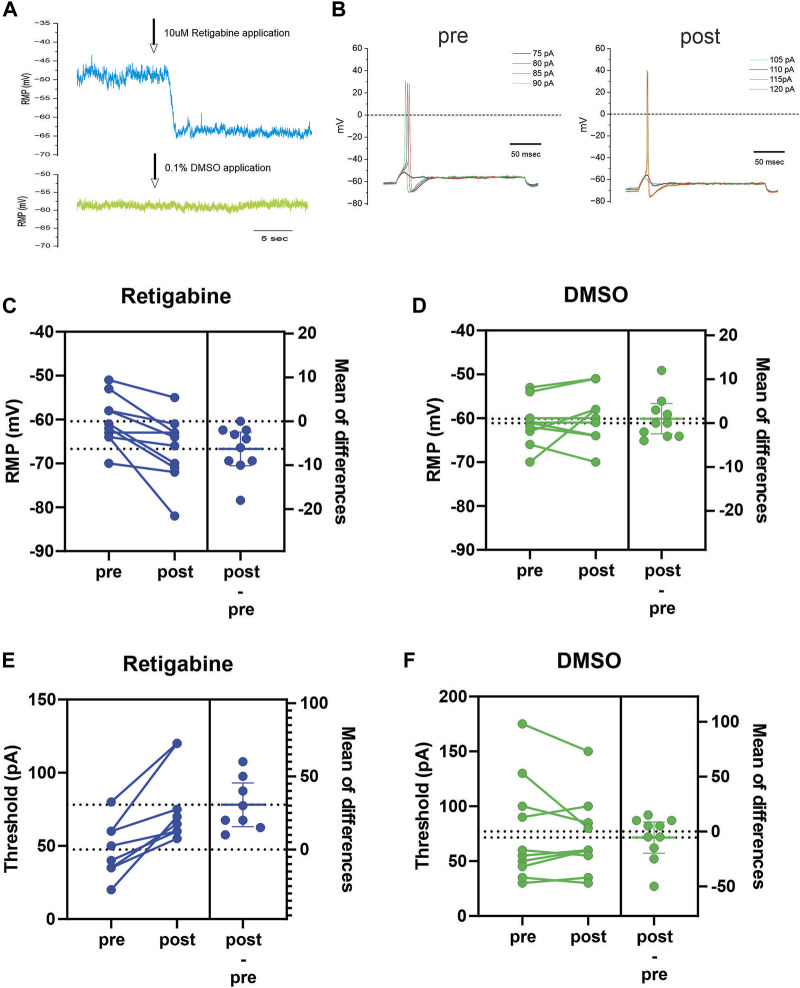
Retigabine hyperpolarizes RMP and increases current threshold **(A)** Example traces showing the hyperpolarizing shift of RMP in response to application of 10 µM retigabine (blue trace). This cell showed a stable RMP of −49 mV during baseline period and a clear shift of RMP to −63 mV in response to retigabine. In a different cell, 0.1% DMSO vehicle control caused little or no change of RMP (green trace). **(B)** Example traces from a representative cell which first fired a spike at 80 pA from a RMP of −61 mV during baseline period and then the RMP shifted to −69 mV in response to 10 µM retigabine and increased the threshold to 115 pA. **(C)** Combined before and after shifts of RMP in response to 10 µM retigabine. Nearly every cell showed a hyperpolarization of RMP and showed a significant average hyperpolarization of 6.3 mV (*n* = 10). **(D)** Combined before and after shifts of RMP in response to vehicle control 0.1% DMSO. The shifts were small and variable and the 1 mV depolarizing shift of RMP was not significant (*n* = 10). **(E)** Combined before and after shifts of threshold in response to 10 µM retigabine. Every cell showed an increase of current needed to elicit a first spike with a statistically significant average increase of 31 pA (*n* = 10). **(F)** Combined before and after shifts of threshold in response to vehicle control 0.1% DMSO. The shifts were small and variable and the 5 pA reduction of threshold was not significant (*n* = 10).

Finally, we used current-clamp analysis to assess the actions of retigabine at the membrane level. Activation of Kv7 channels is expected to impact neuronal excitability by modulating at least two parameters that can be measured in current-clamp mode. Increased Kv7 current would be expected to cause a hyperpolarization of resting membrane potential (RMP) as well as an increase of current stimulus to reach threshold to first action potential. We performed current-clamp recordings from iPSC-SN clone 129 to evaluate the effects of retigabine, holding cells long enough to carry out an analysis before-and-after introduction of the Kv7 modulator. As predicted, 10 µM retigabine consistently hyperpolarized RMP in these human-derived sensory neurons (Figure 4). Examples of the response of RMP to either retigabine (Figure 4A, blue trace) or DMSO (Figure 4A, green trace) are shown. With the perfusion system utilized, a clear hyperpolarizing shift of RMP was seen after a few seconds of lag that stabilizes to a new stable RMP level. For a representative cell exposed to 10 µM retigabine, example traces are shown that illustrate the change of current threshold (Figure 4B). For this dataset, the shift of RMP in response to retigabine was significant with an average shift of −6.3 ± 1.7 mV (*p* < 0.005, *n* = 10), while the average shift of 1 ± 1.6 mV (*p* = 0.53, *n* = 10) in response to 0.1% DMSO vehicle control was not significant (Figures 4C, D). These same cells also showed consistent increase of current threshold in response to retigabine which was not seen to vehicle control. The average increase of current threshold in response to retigabine was significant at 31 ± 6 pA (*p* < 0.002, *n* = 10) whereas the small −6 ± 6 pA (*p* = 0.4, *n* = 10) shift in response to DMSO did not reach significance (Figures 4E, F).

## 4 Discussion

The ability of an excitable cell to fire an action potential requires the coordinated expression and activity of multiple voltage-gated ion channels (Waxman, 2012). Voltage-gated sodium channels enable transient influx of positively charged sodium ions into the cell which leads to depolarization of the membrane potential which generates a positive feedback loop to open further voltage-gated sodium channels to underlie the all or nothing upstroke phase of the action potential. The membrane depolarization also leads to the opening of voltage-gated potassium channels and the resulting outward flux of potassium ions works to repolarize the membrane potential back towards a stable resting membrane potential. Numerous kinds of voltage-gated sodium and potassium channels, each with its own voltage ranges of gating and varying kinetics of voltage response, lead to profoundly non-linear behavior of cellular excitability which makes determining the impact of modulating subsets of these ion channels with pharmacological agents challenging (Cannon and Miller, 2017; Ratliff et al., 2021). On the basis of our finding of gain-of-function variants of Kv7.2 (Mis et al., 2019) and Kv7.3 (Yuan et al., 2021) in pain-resilient subjects and our demonstration that these Kv7 variants reduced excitability of iPSC-SN derived from these subjects, we hypothesized that pharmacological activators of Kv7.2 and Kv7.3 might reduce excitability of iPSC-SN from a subject (P129) from the same family with IEM and the F1449V mutation in Nav1.7 (Dib-Hajj et al., 2005) who did not carry Kv7.2 or Kv7.3 variants (Yuan et al., 2021). We show here that both the Kv7 activator retigabine and the Kv7.2/Kv7.3 selective compound ICA-110381 are effective at inhibiting spontaneous spiking activity whereas the Kv7.1 selective compound ML277, studied as a comparator, was ineffective at inhibiting spiking activity from these iPSC-SN. This profile is consistent with the reports that DRG neurons express high levels of Kv7.2/Kv7.3 while expressing little or no Kv7.1 channels (Passmore et al., 2003; Brown and Passmore, 2009; Barrese et al., 2018) and that our patient-derived iPSC-SN represent a good model system for *in vitro* studies (Alsaloum et al., 2022; Alsaloum and Waxman, 2022).

The use of multi-well MEA platforms is an enabling assay that allows for non-invasive monitoring of spontaneous spiking activity and the ability to simultaneously compare different treatments at the same time. The environmental control ability of the multi-well MEA platform also improves the assay of sensory neurons because they profoundly increase their spontaneous activity when recorded at physiological (compared to room) temperatures which improves the ability to resolve the effects of compounds predicted to reduce excitability. To overcome well-to-well variability in spiking rates that can vary over a 10–100-fold range, we have implemented an approach that transforms the spike counts to the Log10 spike counts which enable the comparison of MEA data (Negri et al., 2020) in a statistically amenable manner. After the logarithm transform, we created subgroups of wells that share similar mean and standard deviation values, providing a platform for multi-concentration compound response experiments that appear to form standard binding curves. The IC_50_ parameter in this case represents the concentration of compound that reduces the activity halfway between the basal (maximal) firing and the total cessation (minimum) firing activity. In terms of fitting the IC_50_ parameter reported here with literature results, we would note that in spiking data not transformed to the logarithm, a 50% reduction of activity would map to a 0.3 reduction of the mean value expressed on the Log10 scale. Thus for our retigabine dose-response shown in Figure 1, the upper fit value of the fitted curve is 3.7 which represents an average total spike count of 5012. A reduction of the curve to 3.4 (spike count of 2510) corresponds to a retigabine concentration of just 0.6 µM. The fitted IC_50_ value of 1.7 µM occurs at the log10 value of 1.86 (spike count of 72). Thus the IC_50_ concentrations reported here may be shifted higher than a corresponding IC_50_ obtained using methods not based on the Log10 transform. For example, the concentration-response relationships for shifting the activation voltage-dependence was found to vary depending on the Kv7 isoform examined with channels encoded by KCNQ3 responding with an EC_50_ of 0.6 µM, KCNQ2/3 channels responding with an EC_50_ of 1.9 µM and channels encoded by KCNQ4 responding with an EC_50_ of 5.2 µM (Tatulian et al., 2001). Assuming that the dominant isoform expressed in our iPSC-SN is Kv7.2/3, then our IC_50_ concentration reported here and the EC_50_ for shifting activation voltage-dependance are in good agreement.

Our previous studies utilizing these patient-derived iPSC-SN suggest that significant pain resilience is associated with reductions of excitability of 50% or less (Mis et al., 2019; Yuan et al., 2021). The concentration-response curve from our MEA assay suggests that retigabine can achieve a 50% reduction of spontaneous firing with sub-micromolar concentrations. Prior studies that have assessed the effect of retigabine in pain models in many cases have assessed only a single dose or a small number of concentrations in the 1–10 µM range (Roza and Lopez-Garcia, 2008; Xu et al., 2010; Du et al., 2014; Hayashi et al., 2014; Abd-Elsayed et al., 2015; Li et al., 2019; Zhang et al., 2020). Taken together, this study and previous results implicate Kv7.2/Kv7.3 channels as effective modulators of sensory neuron excitability and suggest that compounds that specifically target Kv7.2/Kv7.3 currents in sensory neurons, including human sensory neurons, might provide an effective approach toward pain relief.

Retigabine was originally FDA approved as an anti-epileptic which significantly reduced seizures in patients (Orhan et al., 2012). The target for these effects of retigabine were determined to be the Kv7 family of voltage-gated potassium channels (Tatulian et al., 2001; Gunthorpe et al., 2012) and the mechanism of action was reported to be a compound-induced leftward shift of the voltage-dependence of activation resulting in both channels that are easier to open in response to membrane depolarizations as well as increased basal activity since the shifted activation voltage-dependence results in an increased fraction of channels that are open at resting membrane potential (Tatulian et al., 2001; Wang et al., 2018). The use of retigabine, however, has been restricted due to significant side-effects (Faulkner and Burke, 2013; Clark et al., 2015), including the appearance of blue discoloration in tissues, especially the skin and the eyes. Chemically related compounds that maintain the effects of retigabine while avoiding some of these side effects are being developed (Wua and Dworetzky, 2005; Wu et al., 2013). Since retigabine modulates Kv7.2 thru Kv7.5, Retigabine will also impact tissues such as smooth muscle which could lead to hypotension or defects in lymphatic function (Jepps et al., 2013; Khanamiri et al., 2013) or skeletal muscle where retigabine is reported to modulate proliferation and differentiation (Iannotti et al., 2010). Compounds that activate Kv7.2/Kv7.3 channels in a specific manner might show efficacy against peripheral sensory neurons while having a smaller effect on tissues such as smooth muscle which predominantly express Kv7.1, Kv7.4, and Kv7.5 isoforms. Thus a compound with isoform selectivity properties similar to the ICA-110381 would be expected to show profound pain relief without sharing some of the off-target side-effects of retigabine. Of course very high selectivity differentiating between different related subtypes is very difficult to achieve. Additionally, the documented ability for Kv7 subtypes to form heteromeric channels also makes avoiding off-target effects in alternate tissues very challenging (Miceli et al., 2008; Jepps et al., 2013; Barrese et al., 2018). Local application of the drug might also allow the compound to reach efficacious concentrations close to sites of impulse generation in the periphery while minimizing systemic concentrations of drug that might cause off-target side effects. The results of the present study show that Kv7.2/7.3 modulators reduce the firing of sensory neurons derived from human iPSCs, including iPSCs derived from human subjects with severe chronic pain, and underscore the importance of further study of these compounds as a prelude to clinical studies on patients with pain.

## Data Availability

The datasets for this study can be found at DataDryad.org [dataset "Estacion Kv7 modulators of hiPSC-SN" (doi:10.5061/dryad.1jwstqk01)]
